# Biological Effectiveness of Ion Beam for *In Vitro* Cell Irradiations

**DOI:** 10.3389/fonc.2022.847090

**Published:** 2022-06-29

**Authors:** Heng Li

**Affiliations:** Department of Radiation Oncology and Molecular Radiation Sciences, Johns Hopkins University, Baltimore, MD, United States

**Keywords:** ion beam irradiation, radiobiologic modeling, radiotherapy, cell irradiation, modeling

## Abstract

Despite numerous ion beam irradiation of cell experiments performed over the past five decades, the relationship between the biological effectiveness of ion beams and the physical characteristics of the ion beam remains unclear. Using 1,118 sets of *in vitro* cell survival experiments with ion beam irradiation, compiled by the Particle Irradiation Data Ensemble (PIDE) project, the relationship between cell survival and the fluence and linear energy transfer (LET) of the ion beam was established. Unlike previous studies, the closed-form analytical function is independent of photon irradiation and takes a universal form across all ion and cell species. A new understanding of the biological effectiveness of ion beams is crucial for predicting tumor response and toxicities in ion beam radiation therapy, along with radiation protection for high-LET ion beams with low fluence.

## Introduction

Ion beam radiation has a different biological effectiveness compared to photon radiation, manifesting as different cell-killing effects for *in vitro* cell irradiation experiments (1). In practice, the ion beam’s biological effectiveness is relative to the referencing photon beam irradiation [relative biological effectiveness (RBE)]. Numerous experiments have been performed over the past five decades (2), and it has been well established that the biological effectiveness of the ion beams is determined by both the cell properties, including cell type, cell cycle (3, 4), and oxygen level (5, 6), and the physical characteristics of the ion beam, such as ion type, beam energy, linear energy transfer (LET, *L*_∞_ ), fluence (Φ), or dose (D) (1, 7). Experimental data from each study are extremely sparse compared to the wide range of cell and ion beam variations. To enable systematic characterization of the biological effectiveness of ion beams, with the specific endpoint of clonogenic cell survival (8), the Particle Irradiation Data Ensemble (PIDE) project (2) compiled 1,118 sets of *in vitro* cell survival experiments from irradiation results drawn from 115 publications. **Supplement 1** shows the complete list of these publications.

By analyzing the PIDE data, a common close form function of fluence and LET of the ion beam was established for the first time to describe the survival fractionation (SF) of cells for all ion beam experiments.

## Materials and Methods

### Dose, Fluence, and LET

Absorbed dose is defined as the amount of energy imparted to matter by ionizing radiation per unit mass of the matter. It could be represented as a product of fluence and LET:


(1) $$D\left(\text{Gy}\right)=\frac{dE}{dm}=\frac{\Phi L_{\infty }}{\rho }=1.602\times 10^{-1}\times \Phi \left(1/\mu {\text{m}}^{2}\right)\times L_{\infty }\left(\text{keV/}\mu \text{m}\right)\times 1/\rho \left({\text{cm}}^{3}\text{/g}\right)$$

In this equation, Φ is the fluence of the ion beam, *ρ* is the density of the medium, and *L*_∞_ is the unrestricted LET, defined as the energy loss per unit distance, and is the same as stopping power (9). The constant 1.602 × 10^-1^ is for the conversion of units, including eV to Joules. For this study, *ρ* could be safely assumed to be water with unity density. Absorbed dose, fluence, and LET are all measurable physical quantities.

### PIDE Data Elements

The PIDE project compiles 1,118 sets of ion beam irradiation from 115 publications between 1965 and 2015 (please see **Supplement 1** for the complete list of the publications). The data elements collected by PIDE include experiment specifications, such as the investigated cell lines, radiation qualities used, and delivery techniques. LET, α, β, cell line, and ion type for *in vitro* cell irradiation experiments as compiled by the PIDE project were used for this study. In addition, the raw data, or dose and SF, for 962 out of 1,118 sets of experiments were also available through the PIDE project. The number of experiments for different cell lines and ions is summarized in **Table 1**.

**Table 1:** The number of experiments compiled in the PIDE project presented for a subset of ions and cell lines with more than 15 sets of experiments.

	Total	V79	V79 asynchronous cell phase	T1	HSG	NB1RGB	C3H10T1/2	CHO	B14FAF28	R-1	HF19	AG01522	SQ20B	Other cell lines
^1^H	180	52	50				8				3	14	3	100
^2^H	17	10	4				5							2
^3^He	44	31	23		12		1							
^4^He	107	36	26	7			7				10	2		45
^12^C	385	70	61	22	21	24	2	14		11	4	3	11	203
^20^Ne	131	23	20	23	21	15	1	9		11		2		26
^40^Ar	57	21	17	11			1	1	6	6		1	1	9
Other ions	197	77	77	6		12	9	10	24		8			51
Total	1,118	320	278	69	54	51	34	34	30	28	25	22	15	436

^1^H, hydrogen ion with a mass number of 1 (proton). Empty cells represent that no experiments were performed using the combination of ion and cell lines.

### Generation of Dose vs. SF Curves and Fluence–LET–SF Surface

Many biophysical models have been proposed to describe the relationship between absorbed dose and cell survival. A comprehensive review of such models is beyond the scope of the study but can be found in Hall and Giaccia (10). The linear-quadratic (LQ) model (11), which is the most commonly used model, takes the following form:


(2) $$SF=e^{-\left(\alpha D+\beta D^{2}\right)}$$

where parameters are usually determined by experiments and vary with cell type and type of radiation; this relationship holds for ion beam irradiations, and mechanistic modeling of cellular survival linked the radiation-induced DNA double-strand breaks to *α* and *β* of the LQ model (12). In ion beam irradiation, where LET is generally fixed, the model describes the relationship between ion beam fluence and cell survival. As mentioned above, *α* and *β* were reported for all experiments compiled in PIDE, and thus, the dose vs. SF curve could be derived using equation 2.

LET is also reported for each set of experiments compiled in PIDE. Thus, the fluence could be calculated with equation 1 using dose and LET and thus create the fluence–LET–SF surface.

### Fluence–LET Fitting on an Iso-Survival Plane

The fluence–LET combination needed to achieve a specific SF can be found from the fluence–LET–SF surface. To better illustrate the roles of fluence, LET, and SF in ion beam irradiation, the published data from Weyrather etal. (13) were used as an example. In Weyrather et al., 21 sets of cell irradiation experiments were performed with carbon ion beams (^12^C) of various LET ranging from 13.7 to 482.7 keV/μm. The experiments were carried out for three cell lines, V79 (8 sets), CHO-K1 (7 sets), and xrs5 (6 sets). In each experiment set, cells were irradiated with a carbon ion beam of a certain LET to various dose levels. The cell survival fractionation was determined through the experiments, thus establishing the relationship between dose and SF under a fixed LET using the LQ model. The cell SF as a function of dose and linear-quadratic (LQ) fit parameters, *α* and *β*, were reported for each cell line and ion beam with distinctive LET.

**Figure 1:**
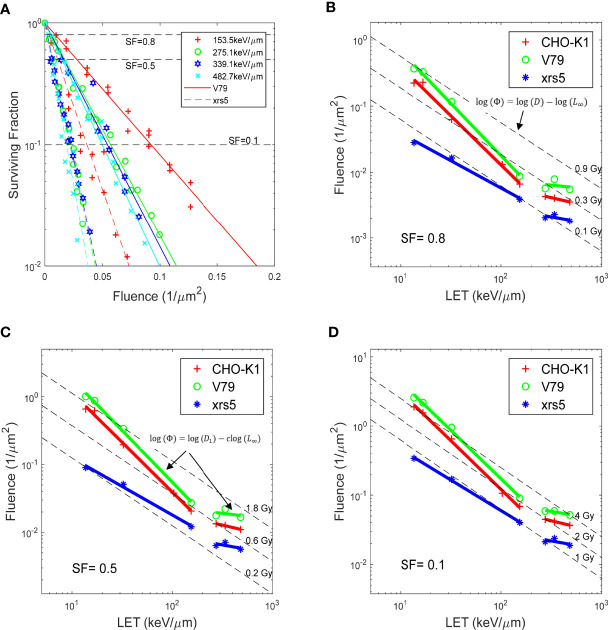
**(A)** Fluence vs. SF for V79 and xrs5 cells, for carbon irradiation with different LET. The same marker represents the same LET carbon irradiation, solid and dash lines are the fitted response for V79 and xrs5 cells, respectively. **(B-D)** show at survival fractionation of 0.8, 0.5 and 0.1, respectively, the fluence needed for various LET beams to achieve the survival fractionation, for three different cell lines. Also plotted are relevant dose levels for the corresponding survival fractionations. Data extracted from Weyrather et. al. (13).

These figures were recreated with the reported α and β, and compared against the raw data for the 21 sets of experiments acquired through the PIDE project. **Figures 1**, **2** in Weyrather et al. showed fluence vs. SF for various cell lines irradiated by carbon beams of various LET, without further discussion. The fluence vs. SF plots for carbon ion beams with LET greater than 150 keV/μm for V79 and xrs5 cell lines in the Weyrather study were recreated in **Figure 1A**. It can be observed that the fluence required to achieve the same SF becomes almost the same for the higher LET beams in each cell line. In other words, the same number of ions leads to the same amount of cell death, or the cell-killing cross-section of different high-LET (over 150 keV/μm) ion beams becomes almost constant (5). LET, fluence, and SF form a surface with SF determined for all LET and fluence values. To better illustrate the relationship, the LET and fluence needed to achieve the SF levels of 0.8, 0.5, and 0.1, as indicated by dashed lines in **Figure 1A**, for each set of the experiments, are shown in **Figures 1B–D** with log–log scale. Each data point on the plot represents the LET/fluence combination required to achieve the SF for different cell lines and carbon ion beams with different LET. It immediately became evident that for each cell line, the data points fell on two distinct lines, above and below LET of 150 keV/μm. As the product of LET and fluence, the same dose could also be represented as a line on the LET–fluence map, which is shown in **Figures 1B–D** as dashed lines. The fluence–LET combination required to achieve a specific SF deviates from the iso-dose lines. In other words, the same dose does not result in the same cell survival in experiments with different LET ion beams.

**Figure 2:**
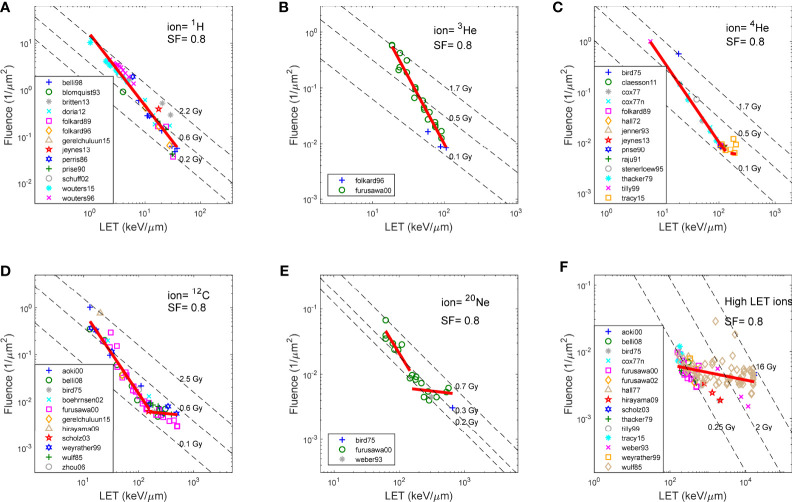
Fluence and LET required to achieve SF of 0.8, for V79 cells in asynchronized cell cycle using various ions, as reported in literature. **(A)** 1H (proton),**(B)** 3He, **(C)** 4He, **(D)** 12C, **(E)** 20Ne, and **(F)** High LET ions (>150 keV/μm). Note that the same fitting in **(F)** was applied to high LET region of the plots in **(C-E)**.

When plotted on a log–log scale, equation 1 becomes:


(3) $$\text{log}\left(\Phi \right)=\text{log}\left(D\right)-\text{log}\left(L_{\infty }\right)$$

as shown by the dashed lines in **Figures 1B–D**. However, the lines that better describe the relationship between fluence and LET are:


(4) $$\text{log}\left(\Phi \right)=\text{log}(D_{1})-c\text{log}\left(L_{\infty }\right)$$

where *D*_1_, which describes the intersection between the line and LET of 1 keV/μm, and *c*, which describes the slope of the line, could be determined for each SF using a linear fit. The relationship among SF, LET, and fluence can then be rewritten as:


(5) $$SF=e^{-aD_{1}^{b}}$$

(6) $$D_{1}=\Phi L_{\infty }^{c}$$

where parameters *a* and *b* vary with cell properties and ion type; *c* varies with cell properties and ion type, along with Φ and *L*_∞_. *c* and *D*_1_ are both bijective functions of SF, and *D*_1_ is numerically equal to the dose required to achieve SF for a particular ion type with a LET of 1 keV/μm.

With equations 5 and 6, it is straightforward to calculate RBE for any ion type with a given SF:


(7) $$RBE_{type}\left(SF\right)=\frac{D_{ref}}{D_{type}}=\frac{D_{ref}\left(SF\right)}{\Phi \;\left(SF\right)L_{\infty }}=\frac{D_{ref}\left(SF\right)}{D_{1}\left(SF\right)}{\left(L_{\infty }\right)}^{c-1}$$

where *D*_ref_(SF)/*D*_1_(SF) is a bijective function of SF.

### Biological Effect Models

To date, the constant 1.1 RBE model is still the clinical standard for proton radiotherapy, where the RBE of a proton beam is determined by:


(8) $$RBE_{proton}=1.1$$

There is also a consensus that proton RBE increases approximately linear with LET up to 10–15 keV/μm (14). In this study, a generic LET linear model was investigated:


(9) $$RBE_{proton}=c_{0}+c_{1}\cdot LET$$

where *c*_0_ and *c*_1_ were determined using the best fit of the same PIDE data for proton irradiation of V79 cells at asynchronized cell cycle, at an SF of 0.5. The resulting *c*_0_ and *c*_1_ are 0.923 and 0.077, respectively. These numbers are close to reported numbers in the literature (15, 16).

The constant, LET linear, and the LET power models derived in this study were calculated for each data point in the PIDE proton V79 data. The normalized root-mean-square error (NRMSE) between the experimental data and models was calculated.


(10) $$NRMSE=\sqrt{\frac{\sum_{t=1}^{T}{\left({\hat{X}}_{t}-X_{t}\right)}^{2}}{T}}/\left(X_{max}-X_{min}\right)$$

where *T* is the size of the sample, *X* is the measurement, and $\hat{X}$ is the model estimation and normalized by the range of the measurements.

## Results

**Figure 2** shows the fluence and LET required to achieve an SF of 0.8, for V79 cells in asynchronized cell cycle using various ions, as reported in the literature over the years and compiled by the PIDE project. **Figures 2A–E** show ^1^H (proton), ^3^He, ^4^He, ^12^C, and ^20^Ne, respectively, and **Figure 2F** shows all high-LET ions (>150 keV/μm, other than ^4^He, ^12^C, and ^20^Ne). Specifically, V79 irradiation data from Weyrather et al. are shown in **Figure 2D** as hexagons. The linear fit of the data is shown as solid red lines in each figure, whereas iso-dose lines are shown as dashed lines. Note that the same fitting in **Figure 2F** was applied to the high-LET region of the plots in **Figures 2C–E**. It is demonstrated in the figures that for the same cell line, the fluence–LET needed to achieve an SF of 0.8 for all ion beams deviates from the dose lines. Instead, the relationship between fluence and LET follows a line with slope *c*, where *c* varies for different SFs, as illustrated in **Figures 1B–D**.

**Figure 3:**
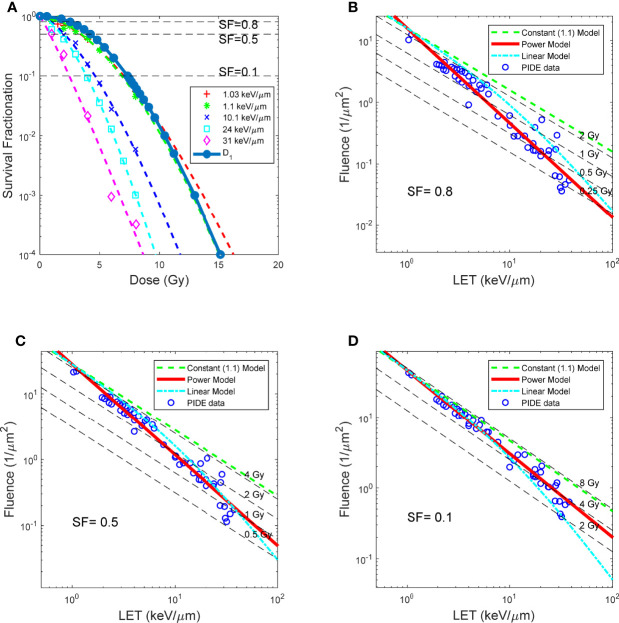
BE as a function of LET, for asynchronized V79 cell irradiations with proton. **(A)** Dose response curves of protons with different LET, and survival as a function of D1. **(B–D)** LET and fluence needed for proton beams to achieve SF 0.8, 0.5 and 0.1, respectively. RBE models prediction overlay with measured data. Red solid lines: RBE calculated using LET power model (Eq. 7). Green dash lines: constant (1.1) RBE model (Eq. 8). Cyan dash lines: linear RBE model (Eq. 9). Data points: RBE calculated using reported experiment results complied in PIDE, total of 278, detailed in **Table 1**.

Subsequently, for each cell line, ion type, and SF, parameters *D*_1_ and *c* could be determined by linear fitting. The resulting *D*_1_ for V79 cells in asynchronized cell cycle using proton beams to achieve different SFs is shown in **Figure 3A**, where *D*_1_ vs. SF is plotted along dose vs. SF using proton beams of varying LETs ranging from 1 to 31 keV/μm. As shown in the figure, *D*_1_ closely resembles the dose–response curves of proton beams with 1.03 and 1.1 keV/μm. It is worth noting that LET for heavier ions is always higher than 1 keV/μm, and as such, *D*_1_ is just a value for reference purposes.

**Figures 3B–D** compared the constant 1.1 model, a generic LET linear model, and the LET power model shown in this study. It could be observed that the LET power model correlates well with experimental data on all three SF levels. In contrast, the LET linear model correlates reasonably well with the experimental data at lower LET but trends away from the experimental data with increasing LET. Finally, the constant model overestimates the dose required to achieve a specific SF, thus underestimating the biological effectiveness of the proton beam, especially for high-LET proton beams. The NRMSE of the three models compared with the experimental data is summarized in **Table 2**.

**Table 2:** Experimental data for PIDE proton beam irradiation of V79 cell line in asynchronous cell phase.

	SF	Constant (1.1) RBE model	LET Linear RBE model	LET Power RBE model
NRMSE	0.8	0.1498	0.1160	0.0555
	0.5	0.1137	0.0802	0.0319
	0.1	0.0705	0.0532	0.0271

Normalized root-mean-square error (NRMSE) between the experimental data and constant (1.1) model, LET linear model, and LET power model, at SF levels of 0.8, 0.5, and 0.1.

## Discussion

Radiation induces ionization events, which cause DNA damage, including double-strand breaks (DSB) and clustered damages, leading to cell death (17, 18). Dose quantifies the energy departed, thus quantifying the total number of ionization events. However, by definition, dose only describes the total energy imparted and offers no information on the spatial distribution of the energy deposition in the form of ionization events. For photon irradiation, the lack of spatial information of dose is not a huge problem as the energy deposition by photon radiation could, in general, be considered homogeneous. However, ionization events are much denser around ion tracks for ion beams. The dose is no longer adequate to be used as the sole parameter to quantify the biological effects of ion beams. It became clear that, unlike photon irradiation, the spatial distribution of the energy deposition plays an essential role in the biological effectiveness of the ion beam.

In contrast, LET is the energy loss per unit length and could be considered a measurement of the density of ionization events around ion tracks, whereas fluence represents the number of ion tracks per unit area. Thus, both fluence and LET could provide additional insight into the spatial distribution of ionization events and energy deposition.

In place of dose, cell survival is better described by different quantities, namely, fluence and LET, which are closely related but distinctly different from dose. Additionally, equations 5 and 6 are independent of any photon irradiation and thus represent the biological effectiveness instead of the RBE of the ion beams.

In equations 5 and 6, the parameter *c* describes the relative importance of fluence and LET in introducing damages that lead to cell death, where, for dose, in the form of the product of fluence and LET, there is an implicit assumption that the relative contribution from fluence and LET is equal (*c* = 1). In general, *c* is higher for higher SF (less cell killing), which means that increasing LET is more effective than increasing fluence to achieve the same SF. For lower SF (more cell killing), *c* is closer to unity as the spatial distribution of the ionization events becomes less critical. *D*_1_ represents the intersection point between the fluence–LET line and a LET of 1 keV/μm, and numerically equals the dose needed to achieve the specific SF using an ion beam with 1 keV/μm, based on equation 5. As shown in **Figure 3A**, *D*_1_ closely resembles the dose–response curves of proton beams with 1.03 and 1.1 keV/μm.

One of the study’s fundamental limitations is that the dose, LET, or fluence required to achieve a certain SF could not be directly measured. Instead, it has to be interpolated through modeling of the measured data. The accuracy of the model, specifically the LQ model, thus directly impacts the accuracy of the downstream analyses. Additionally, while the study considers the beam characteristics, including ion type, ion energy, LET, and fluence, and cell characteristics, including cell type and phases of the cell cycle, there are still other factors, such as oxygen concentration and dose rate, that could have an impact on the cell survival. These factors need to be investigated in follow-up studies.

With the recent advance in proton radiotherapy, a number of biological effect models for proton beams have been proposed and evaluated (19). To date, the constant 1.1 model is still the clinical standard for proton radiotherapy, whereas a consensus that proton RBE increases approximately linear with LET up to 10–15 keV/μm was reached (14). Our study demonstrated that the power model could describe the biological effect better than both the constant 1.1 and LET linear models. Since both LET and fluence are readily available in current Monte Carlo dose engines, the biological effect model could easily be implemented for treatment planning. However, before any attempt at clinical implementation, it would be necessary to evaluate and validate the model with clinical outcome using retrospective data.

## Conclusion

In summary, dissecting the biological effectiveness of ion beams as a function of fluence and LET describes the existing data better than using only dose, which is a derived function of the two quantities. Using cell survival as an endpoint, the biological effectiveness of all ion types could be described as a common function of fluence and LET. For ions above a particular LET, further increasing LET without increasing fluence is not effective in increasing cell killing. The model needs to be evaluated and validated using clinical data.

## Conflict of Interest

The author declares that the research was conducted in the absence of any commercial or financial relationships that could be construed as a potential conflict of interest.

## Publisher’s Note

All claims expressed in this article are solely those of the authors and do not necessarily represent those of their affiliated organizations, or those of the publisher, the editors and the reviewers. Any product that may be evaluated in this article, or claim that may be made by its manufacturer, is not guaranteed or endorsed by the publisher.

## Data Availability Statement

The data used in this study is available through PIDE. https://www.gsi.de/work/forschung/biophysik/forschungsfelder/radiobiological_modelling/pide_project. further inquiries can be directed to the corresponding author.

## Author Contributions

HL is responsible for the study.

## Acknowledgments

I acknowledge the PIDE project for compiling available ion beam *in vitro* cell irradiation data.

## Supplementary Material

Supplement 1 Full list of PIDE references.
