# Supplementary material for: Biological Effectiveness of Ion Beam for In Vitro Cell Irradiations
Source: Front Oncol. 2022 Jun 29;12:847090. doi: 10.3389/fonc.2022.847090 (PMC9278697; doi:10.3389/fonc.2022.847090)
Supplement: Supplement 1 — Full list of PIDE references. [file DataSheet_1.pdf]

Particle Irradiation Data Ensemble: References

[1] Chapman JD, Blakely EA, Smith KC *et al*. Radiation biophysical studies with mammalian cells and a modulated carbon ion beam. *Radiat Res* 1978; **74**:101–11.

[2] Chapman JD, Blakely EA, Smith KC *et al*. Radiobiological characterization of the inactivating events produced in mammalian cells by helium and heavy ions. *Int J Radiat Oncol Biol Phys* 1977; **3**:97–102.

[3] Blakely EA, Tobias CA, Yang TCH *et al*. Inactivation of human kidney cells by high-energy monoenergetic heavy-ion beams. *Radiat Res* 1979; **80**:122–60.

[4] Raju MR, Eisen Y, Carpenter S *et al*. Radiobiology of α particles. *Radiat Res* 1991; **128**:204–9.

[5] Goodhead DT, Belli M, Mill AJ *et al*. Direct comparison between protons and alpha-particles of the same LET. I: irradiation methods and inactivation of asynchronous V79, HeLa and C3HT1/2 cells. *Int J Radiat Biol* 1992; **61**:611–24.

[6] Folkard M, Prise KM, Voijnovic B *et al*. Inactivation of V79 cells by low-energy protons, deuterons and helium-3 ions. *Int J Radiat Biol* 1996; **69**:729–38.

[7] Eguchi-Kasai K, Murakami M, Itsukaichi H *et al*. The role of DNA repair on cell killing by charged particles. *Adv Space Res* 1996; **18**:109–18.

[8] Suzuki M, Kase Y, Kanai T *et al*. LET dependence of cell death and chromatin-break induction in normal human cells irradiated by neon-ion beams. *Int J Radiat Biol* 1997;**72**:497–503.

[9] Bettega D, Calzolari P, Marchesini R *et al*. Inactivation of C3H10T1/2 cells by low energy protons and deuterons. *Int J Radiat Biol* 1998; **73**:303–9.

[10] Tsuboi K, Tsuchida Y, Nose T *et al*. Cytotoxic effect of accelerated carbon beams on glioblastoma cell lines with p53 mutation: clonogenic survival and cell-cycle analysis. *Int J Radiat Biol* 1998; **74**:71–9.

[11] Tsuchida Y, Tsuboi K, Ohyama H *et al*. Cell death induced by high-linear-energy transfer carbon beams in human glioblastoma cell lines. *Brain Tumor Pathol* 1998; **15**:71–6.

[12] Weyrather WK, Ritter S, Scholz M *et al*. RBE for carbon track-segment irradiation in cell lines of differing repair capacity. *Int J Radiat Biol* 1999; **75**:1357–64.

[13] Furusawa Y, Fukutsu K, Aoki M *et al*. Inactivation of aerobic and hypoxic cells from three different cell lines by accelerated 3He-, 12C- and 20Ne beams. *Radiat Res* 2000; **154**:485–96.

[14] Suzuki M, Kase Y, Yamaguchi H *et al*. Relative biological effectiveness for cell-killing effect on various human cell lines irradiated with heavy-ion medical accelerator in Chiba (HIMAC) carbon-ion beams. *Int J Radiat Oncol Biol Phys* 2000; **48**:241–50.

[15] Belli M, Bettega D, Calzolari P *et al*. Inactivation of human normal and tumour cells irradiated with low energy protons. *Int J Radiat Biol* 2000; **76**:831–9.

[16] Tsuruoka C, Suzuki M, Fujitaka K *et al*. LET and ion species dependence for cell killing in normal human skin fibroblasts. *Radiat Res* 2005; **163**:494–500.

[17] Belli M, Bettega D, Calzolari P *et al*. Effectiveness of monoenergetic and spread-out Bragg peak carbon-ions for inactivation of various normal and tumour human cell lines. *J Radiat Res* 2008; **49**:597–607.

[18] Belli M, Cera F, Cherubini R *et al*. RBE-LET relationships for cell inactivation and mutation induced by low energy protons in V79 cells: further results at the LNL facility. *Int J Radiat Biol* 1998; **74**:501–9.

[18b] Belli M, Cera F, Cherubini R *et al*. Inactivation and mutation induction in V79 cells by low energy protons: reevaluation of the results at the LNL facility. *Int J Radiat Biol* 1993; **63**:331–7.

[19] Hall EJ, Bird RP, Rossi HH *et al*. Biophysical studies with high-energy argon ions 2. Determinations of the relative biological effectiveness, the oxygen enhancement ratio, and the cell cycle response. *Radiat Res* 1977; **70**:469–79.

[20] Bird RP, Burki HJ. Survival of synchronized Chinese hamster cells exposed to radiation of different linear-energy transfer. *Int J Radiat Biol* 1975; **27**:105–20.

[20b] Sinclair WK, Morton RA. X-ray sensitivity during the cell generation cycle of cultured Chinese hamster cells. *Radiat Res* 1966; **29**:450–74.

[21] Von Neubeck C. Radiobiological experiments for carbon ion prostate cancer therapy: interplay of normal and tumor cells in co-culture and measurement of the oxygen enhancement ratio. PhD thesis, Technische Universität Darmstadt, 2009.

[22] Perris A, Pialoglou P, Katsanos AA *et al*. Biological effectiveness of low energy protons. I. Survival of Chinese hamster cells. *Int J Radiat Biol* 1986; **50**:1093–101.

[23] Bettega D, Tallone Lombardi L. Physical and radiobiological parameters of proton beams up to 31 MeV. *Nuovo Cim* 1983; **2D**:907–16.

[23b] Bettega D, Birattari C, Bombana M *et al*. Relative biological effectiveness for protons of energies up to 31MeV. *Radiat Res* 1979; **77**:85–97.

[24] Cox R, Thacker J, Goodhead DT. Inactivation and mutation of cultured mammalian cells by aluminium characteristic ultrasoft X-rays. II. Dose-responses of Chinese hamster and human diploid cells to aluminium X-rays and radiations of different LET. *Int J Radiat Biol* 1977; **31**:561–76.

[25] Wouters BG, Lam GKY, Oelfke U *et al*. Measurements of relative biological effectiveness of the 70 MeV proton beam at TRIUMF using Chinese hamster V79 cells and the high precision cell sorter assay. *Radiat Res* 1996; **146**:159–70.

[26] Combs SE, Bohl J, Elsässer T *et al*. Radiobiological evaluation and correlation with the local effect model (LEM) of carbon ion radiation therapy and temozolomide in glioblastoma cell lines. *Int J Radiat Biol* 2009; **85**:126–36.

[27] Kitajima S, Nakamura H, Adachi M *et al*. AT cells show dissimilar hypersensitivity to heavy-ion and X-rays irradiation. *J Radiat Res* 2010; **51**:251–5.

[28] Blomquist E, Russell KR, Stenerlöw B *et al*. Relative biological effectiveness of intermediate energy protons. Comparisons with 60Co gamma-radiation using two cell lines. *Radiother Oncol* 1993; **28**:44–51.

[29] Yang TCH, Craise LM, Mei MT *et al*. Neoplastic cell transformation by heavy charged particles. *Radiat Res* 1985; **104**: S177–S187.

[30] Miller RC, Marino SA, Brenner DJ *et al*. The biological effectiveness of radon-progeny alpha particles. II. Oncogenic transformation as a function of linear energy transfer. *Radiat Res* 1995; **142**:54–60.

[31] Czub J, Banas´ D, Błaszczyk A *et al*. Biological effectiveness of 12C and 20Ne ions with very high LET. *Int J Radiat Biol* 2008; **84**:821–9.

[32] Kamlah F, Hanze J, Arenz A *et al*. Comparison of the effects of carbon ion and photon irradiation on the angiogenic response in human lung adenocarcinoma cells. *Int J Radiat Oncol Biol Phys* 2011; **80**:1541–9.

[33] Aoki M, Furusawa Y, Yamada T. LET dependency of heavy-ion induced apoptosis in V79 cells. *J Radiat Res* 2000; **41**:163–75.

[34] Han ZB, Suzuki H, Suzuki F *et al*. Relative biological effectiveness of accelerated heavy ions for induction of morphological transformation in Syrian hamster embryo cells. *J Radiat Res* 1998; **39**:193–201.

[35] Hamada N, Imaoka T, Masunaga S *et al*. Recent advances in the biology of heavy-ion cancer therapy. *J Radiat Res* 2010; **51**:365–83.

[36] Claesson K, Magnander K, Kahu H *et al*. RBE of α-particles from 211At for complex DNA damage and cell survival in relation to cell cycle position. *Int J Radiat Biol* 2011; **87**:72–84.

[37] Wedenberg M, Lind BK, Toma-Dasu I *et al*. Analytical description of the LET dependence of cell survival using the repairable-conditionally repairable damage model. *Radiat Res* 2010; **174**:517–25.

[38] Miller RC, Brenner DJ, Randers-Pehrson G *et al*. The effects of the temporal distribution of dose on oncogenic transformation by neutrons and charged particles of intermediate LET. *Radiat Res* 1990; **14**:S62–S68.

[38b] Miller RC, Marino SA, Brenner DJ *et al*. The biological effectiveness of radon-progeny alpha particles. II. Oncogenic transformation as a function of linear energy transfer. *Radiat Res* 1995; **142**:54–60.

[39] Tobias CA, Blakely EA, Ngo FQH *et al*. The repair misrepair model of cell survival. In: Meyn RE, Withers HR (eds). Radiation Biology in Cancer Research. New York: Raven press, 1980, 195–230.

[40] Cox R, Masson WK. Mutation, inactivation of cultured mammalian cells exposed to beams of accelerated heavy ions III. Human diploid fibroblasts. *Int J Radiat Biol* 1979; **36**:149–60.

[41] Ito A, Nakano H, Kusano Y *et al*. Contribution of indirect action to radiation-induced mammalian cell inactivation: dependence on photon energy and heavy-ion LET. *Radiat Res* 2006; **165**:703–12.

[42] Tilly N, Brahme A, Carlsson J *et al*. Comparison of cell survival models for mixed LET radiation. *Int J Radiat Biol* 1999; **75**:233–43.

[43] Thacker J, Stretch A, Stephens MA. Mutation, inactivation of cultured mammalian cells exposed to beams of accelerated heavy ions II. Chinese hamster V79 cells. *Int J Biol* 1979; **36**:137–48.

[44] Hirayama R, Ito A, Tomita M *et al*. Contributions of direct and indirect actions in cell killing by high-LET radiations. *Radiat Res* 2009; **171**:212–18.

[45] Hirayama R, Furusawa Y, Fukawa T *et al*. Repair kinetics of DNA-DSB induced by X-rays or carbon ions under oxic and hypoxic conditions. *J Radiat Res* 2005; **46**:325–32.

[46] Curtis SB, Schilling WA, Tenforde TS *et al*. Survival of oxygenated and hypoxic tumor cells in the extended-peak regions of heavy charged-particle beams. *Radiat Res* 1982; **90**:292–309.

[47] Böhrnsen G, Weber KJ, Scholz M. Measurement of biological effects of high-energy carbon ions at low doses using a semi-automated cell detection system. *Int J Radiat Biol* 2002; **78**:259–66.

[48] Fournier C, Scholz M, Weyrather WK *et al*. Changes of fibrosis-related parameters after high- and low-LET irradiations of fibroblasts. *Int J Radiat Biol* 2001; **77**:713–22.

[49] Wulf H, Kraft-Weyrather W, Miltenburger HG *et al*. Heavy-ion effects on mammalian cells: inactivation measurements with different cell lines. *Radiat Res* 1985; **104**: S122–S134.

[49b] Wulf H. Der Einfluss von Schwerionenstrahlen auf das Überleben und Wachstum von Säugetierzellen in vitro. PhD thesis, Technische Hochschule Darmstadt, 1983.

[49c] Kraft G. Biologische Effekte schwerer Ionen. In: zum Winkel K ed. Wirkungssteigerung der Strahlentherapie maligner Tumoren. Springer; Heidelberg, 1987.

[50] Scholz M. Effects of ion radiation on cells and tissues. *Adv Polymer Science* 2003; **162**:95–155.

[50b] Weyrather WK, Ritter S, Scholz M *et al*. RBE for carbon track-segment irradiation in cell lines of differing repair capacity. *Int J Radiat Biol* 1999; **75**:1357–64.

[51] Persson LM, Edgren MR, Stenerlöw B *et al*. Relative biological effectiveness of boron ions human melanoma cells. *Int J Radiat Biol* 2002; **78**:743–8.

[52] Yang TC, Tobias CA. Neoplastic cell transformation by energetic heavy ions and its modification with chemical agents. *Adv Space Res* 1984; **4**:207–18.

[53] Scholz M, Kellerer AM, Kraft-Weyrather W *et al.* Computation of cell survival in heavy ion beams for therapy. The model and its approximation. *Radiat Environ Biophys* 1997; **36**:59–66.

[54] Prise KM, Folkard M, Davies S *et al*. The irradiation of V79 mammalian cells by protons with energies below 2 MeV. Part II. Measurement of oxygen enhancement ratios and DNA damage. *Int J Radiat Biol* 1990; **58**:261–77.

[55] Terato H, Tanaka R, Nakaarai Y *et al*. Quantitative analysis of isolated and clustered DNA damage induced by gamma-rays, carbon ion beams, and iron ion beams. *J Radiat Res* 2008; **49**:133–46.

[56] Suzuki M, Watanabe M, Kanai Y *et al*. LET dependence of cell death, mutation induction and chromatin damage in human cells irradiated with accelerated carbon ions. *Adv Space Res* 1996; **18**:127–36.

[57] Matsumoto Y, Iwakawa M, Furusawa Y *et al*. Gene expression analysis in human malignant melanoma cell lines exposed to carbon beams. *Int J Radiat Biol* 2008; **84**:299–314.

[58] Mehnati P, Morimoto S, Yatagai F *et al*. Exploration of 'over kill effect' of high-LET Ar- and Fe-ions by evaluating the fraction of non-hit cell and interphase death. *J Radiat Res* 2005; **46**:343–50.

[59] Stenerlöw B, Petterson OA, Essand M *et al*. Irregular variations in radiation sensitivity when the linear energy transfer is increased. *Radiother Oncol* 1995; **36**:133–42.

[60] Okayasu R, Okada M, Okabe A *et al*. Repair of DNA damage induced by accelerated heavy ions in mammalian cells proficient and deficient in the non-homologous endjoining pathway. *Radiat Res* 2006; **165**:59–67.

[61] Tsuboi K, Moritake T, Tsuchida Y *et al*. Cell cycle checkpoint and apoptosis induction in glioblastoma cells and fibroblasts irradiated with carbon beam. *J Radiat Res* 2007; **48**:317–25.

[62] Todd PW. Heavy-ion irradiation of human and Chinese hamster cells in vitro. *Radiat Res* 1975; **61**:288–97.

[63] Hall EJ, Gross W, Dvorak RF *et al*. Survival curves and age response functions for Chinese hamster cells exposed to X-rays or high-LET alpha-particles. *Radiat Res* 1972; **52**:88–98.

[64] Takahashi A, Ohnishi K, Tsuji K *et al*. WAF1 accumulation by carbon-ion beam and α-particle irradiation in human glioblastoma cultures cells. *Int J Radiat Biol* 2000; **76**:335–41.

[65] Takahashi A, Matsumoto H, Yuki K *et al*. High-LET radiation enhanced apoptosis but not necrosis regardless of pp53 status. *Int J Radiat Oncol Biol Phys* 2004; **60**:591–7.

[66] Matsuzaki H, Miyamoto T, Miyazawa Y *et al*. Biological effects of heavy ion beam on human breast cancers. *Breast Cancer* 1998; **5**:261–8.

[67] Kronenberg A, Gauny S, Kwoh E *et al*. Comparative analysis of cell killing and autosomal mutation in mouse kidney epithelium exposed to 1 GeV/nucleon iron ions in vitro or in situ. *Radiat Res* 2009; **172**:550–7.

[68] Hamada N, Funayama T, Wada S *et al*. LET-dependent survival of irradiated normal human fibroblasts and their descendants. *Radiat Res* 2006; **166**:24–30.

[69] Zhou G, Wang P, Tao L *et al*. Protective effect of melatonin against low- and high-LET irradiation. *J Radiat Res* 2006; **47**:175–8.

[70] Jenner TJ, deLara CM, O'Neill P *et al*. Induction and rejoining of DNA double-strand breaks in V79-4 mammalian cells following γ- and α-irradiation. *Int J Radiat Biol* 1993; **64**:265–73.

[71] Furusawa Y, Aoki M, Durante M. Simultaneous exposure of mammalian cells to heavy ions and X-rays. *Adv Space Res* 2002; **30**:877–84.

[72] Takahashi A, Ohnishi K, Ota I *et al*. p53–dependent thermal enhancement of cellular sensitivity in human squamous cell carcinoma in relation to LET. *Int J Radiat Biol* 2001; **77**:1043–51.

[73] Bettega D, Calzolari P, Doneda L *et al*. Early and delayed reproductive death in human cells exposed to high energy iron ion beams. *Adv Space Res* 2005; **35**:280–5.

[74] Ibañez IL, Bracalente C, Molinari BL *et al*. Induction and rejoining of DNA double strand breaks assessed by H2AX phosphorylation in melanoma cells irradiated with proton and lithium beams. *Int J Radiat Oncol Biol Phys* 2009; **74**:1226–35.

[75] Hellweg CE, Baumstark-Khan C, Schmitz C *et al*. Carbon-ion-induced activation of the NF-κB pathway. *Radiat Res* 2011; **175**:424–31.

[76] Napolitano M, Durante M, Grossi GF *et al*. Inactivation of C3H 10T1/2 cells by monoenergetic high LET alpha-particles. *Int J Radiat Biol* 1992; **61**:813–20.

[77] Hill MA, Herdman MT, Stevens DL *et al*. Relative sensitivities of repair-deficient mammalian cells for clonogenic survival after α-particle irradiation. *Radiat Res* 2004; **162**:667–76.

[78] Antonelli F, Campa A, Esposito G *et al.* Induction and Repair of DNA DSB as Revealed by H2AX Phosphorylation Foci in Human Fibroblasts Exposed to Low- and High-LET Radiation: Relationship with Early and Delayed Reproductive Cell Death. *Radiat Res* 2015; **183**:417–31.

[79] Bettega D, Calzolari P, Hessel P *et al.* Neoplastic transformation induced by carbon ions. *Int J Radiat Oncol Biol Phys* 2009; **73**:861–8.

[80] Beuve M, Alphonse G, Maalouf M *et al.* Radiobiologic parameters and local effect model predictions for head-and-neck squamous cell carcinomas exposed to high linear energy transfer ions. *Int J Radiat Oncol Biol Phys* 2008; **71**:635–42.

[81] Britten RA, Nazaryan V, Davis LK *et al.* Variations in the RBE for cell killing along the depth-dose profile of a modulated proton therapy beam. *Radiat Res* 2013; **179**:21–8.

[82] Chaudhary P, Marshall TI, Perozziello FM *et al.* Relative biological effectiveness variation along monoenergetic and modulated Bragg peaks of a 62-MeV therapeutic proton beam: a preclinical assessment. *Int J Radiat Oncol Biol Phys* 2014; **90**:27–35.

[83] Cox R, Thacker J, Goodhead DT, Munson RJ. Mutation and inactivation of mammalian cells by various ionising radiations. *Nature* 1977; **267**:425–7.

[84] Folkard M, Prise KM, Vojnovic B *et al*. The irradiation of V79 mammalian cells by protons with energies below 2 MeV. Part I: Experimental arrangement and measurements of cell survival. *Int J Radiat Biol* 1989; **56**:221–37.

[85] Franken NA, ten Cate R, Krawczyk PM *et al*. Comparison of RBE values of high-LET α particles for the induction of DNA-DSBs, chromosome aberrations and cell reproductive death. *Radiat Oncol* 2011; **6**:64.

[86] Frankenberg-Schwager M, Gebauer A, Koppe C *et al*. Single-strand annealing, conservative homologous recombination, nonhomologous DNA end joining, and the cell cycle-dependent repair of DNA double-strand breaks induced by sparsely or densely ionizing radiation. *Radiat Res* 2009; **171**:265–73.

[87] Gerelchuluun A, Hong Z, Sun L *et al.* Induction of in situ DNA double-strand breaks and apoptosis by 200 MeV protons and 10 MV X-rays in human tumour cell lines. *Int J Radiat Biol* 2011; **87**:57–70.

[88] Gerelchuluun A, Manabe E, Ishikawa T *et al.* The major DNA repair pathway after both proton and carbon-ion radiation is NHEJ, but the HR pathway is more relevant in carbon ions. *Radiat Res* 2015; **183**:345–56.

[89] Habermehl D, Ilicic K, Dehne S *et al.* The relative biological effectiveness for carbon and oxygen ion beams using the raster-scanning technique in hepatocellular carcinoma cell lines. *PLoS One* 2014; **9**:e113591.

[90] Kraft GH, Blakely EA, Kraft-Weyrather W *et al*. Super Heavy Ions: Uranium Radiobiology and Physics. *LBL Biology and Medicine Department Annual Report 1983-1984* 1985; 128–37.

[91] Manti L, Campajola L, Perozziello FM *et al.* Development of a low-energy particle irradiation facility for the study of the biological effectiveness of the ion track end. *J Phys: Conf Series* 2012; **373**:012019.

[92] Petrović I, Ristić-Fira A, Todorović D *et al*. Response of a radioresistant human melanoma cell line along the proton spread-out Bragg peak. *Int J Radiat Biol* 2010; **86**:742–51.

[93] Raju MR, Amols HI, Dicello JF *et al.* A heavy particle comparative study. Part I: depth-dose distributions. *Br J Radiol* 1978; **51**:699–703.

[94] Hamada N, Hara T, Omura-Minamisawa M *et al*. Energetic heavy ions overcome tumor radioresistance caused by overexpression of Bcl-2. *Radiother Oncol* 2008; **89**:231–6.

[95] Słonina D, Biesaga B, Swakoń J *et al.* Relative biological effectiveness of the 60-MeV therapeutic proton beam at the Institute of Nuclear Physics (IFJ PAN) in Kraków, Poland. *Radiat Environ Biophys* 2014; **53**:745–54.

[96] Takahashi A, Kubo M, Ma H *et al.* Nonhomologous end-joining repair plays a more important role than homologous recombination repair in defining radiosensitivity after exposure to high-LET radiation. *Radiat Res* 2014; **182**:338–44.

[97] Tracy BL, Stevens DL, Goodhead DT, Hill MA. Variation in RBE for Survival of V79-4 Cells as a Function of Alpha-Particle (Helium Ion) Energy. *Radiat Res* 2015; **184**:33–45.

[98] Wada M, Suzuki M, Liu C *et al*. Modeling the biological response of normal human cells, including repair processes, to fractionated carbon beam irradiation. *J Radiat Res* 2013; **54**:798–807.

[99] Weber KJ, Flentje M. Lethality of heavy ion-induced DNA double-strand breaks in mammalian cells. *Int J Radiat Biol* 1993; **64**:169–78.

[100] Wouters BG, Skarsgard LD, Gerweck LE *et al*. Radiobiological intercomparison of the 160 MeV and 230 MeV proton therapy beams at the Harvard Cyclotron Laboratory and at Massachusetts General Hospital. *Radiat Res* 2015; **183**:174–87.

[101] Yashkin PN, Silin DI, Zolotov VA *et al*. Relative Biological Effectiveness of Proton Medical Beam at Moscow Synchrotron Determined by the Chinese Hamster Cells Assay. *Int J Radiat Oncol Biol Phys* 1995; **31**:535–40.

[102] Fournier C, Zahnreich S, Kraft D *et al.* The fate of a normal human cell traversed by a single charged particle. *Sci Rep* 2012; **2**:643.

[103] Antoccia A, Sgura A, Berardinelli F *et al.* Cell cycle perturbations and genotoxic effects in human primary fibroblasts induced by low-energy protons and X/gamma-rays. *J Radiat Res* 2009; **50**:457–68.

[104] Baggio L, Cavinato M, Cherubini R *et al.* Relative biological effectiveness of light ions in human tumoural cell lines: role of protein p53. *Radiat Prot Dosimetry* 2002; **99**:211–4.

[105] Bird RP, Rohrig N, Colvett RD *et al*. Inactivation of synchronized Chinese Hamster V79 cells with charged-particle track segments. *Radiat Res* 1980; **82**:277–89.

[106] Doria D, Kakolee KF, Kar S *et al.* Biological effectiveness on live cells of laser driven protons at dose rates exceeding 10^9 Gy/s. *AIP Advances* 2012; **2**:011209.

[107] Hei TK, Chen DJ, Brenner DJ, Hall EJ. Mutation induction by charged particles of defined linear energy transfer. *Carcinogenesis* 1988; **9**:1233–6.

[108] Inada T, Kawachi K, Kanai T, Nojiri I. Inactivation of cultured human tumor cells irradiated by cyclotron neutrons and protons. *J Radiat Res* 1981; **22**:143–53.

[109] Jeynes JC, Merchant MJ, Barazzuol L *et al*. "Broadbeam" irradiation of mammalian cells using a vertical microbeam facility. *Radiat Environ Biophys* 2013; **52**:513–21.

[110] Ogata T, Teshima T, Kagawa K *et al.* Particle irradiation suppresses metastatic potential of cancer cells. *Cancer Res* 2005; **65**:113–20.

[111] Petrovic I, Ristić-Fira A, Todorović D *et al.*, Radiobiological analysis of human melanoma cells on the 62 MeV CATANA proton beam. *Int J Radiat Biol* 2006; **82**:251–265.

[112] Ristić-Fira A, Todorović D, Žakula J *et al*. Response of Human HTB140 Melanoma Cells to Conventional Radiation and Hadrons. *Physiol Res* 2011; **60(Suppl. 1)**: S129–S135.

[113] Schuff JA, Policastro L, Durán H *et al*. Relative biological effectiveness measurements of low energy proton and lithium beams on tumor cells. *Nucl Instr Meth Phys Res B* 2002; **187**:345–53.

[114] Williams JR, Gould RG, Flynn D *et al.* Relative survival of hybrid X-ray-resistant, and normally sensitive mammalian cells exposed to X rays and protons under aerobic and hypoxic conditions. *Radiat Res* 1978; **73**:585–90.

[115] Guan F, Bronk L, Titt U *et al.* Spatial mapping of the biologic effectiveness of scanned particle beams: towards biologically optimized particle therapy. *Sci Rep* 2015; **5**:9850.
